# Characterizing DNA Condensation and Conformational Changes in Organic Solvents

**DOI:** 10.1371/journal.pone.0013308

**Published:** 2010-10-11

**Authors:** Fuyou Ke, Yen Kim Luu, Michael Hadjiargyrou, Dehai Liang

**Affiliations:** 1 Beijing National Laboratory for Molecular Sciences and the Key Laboratory of Polymer Chemistry and Physics of Ministry of Education, College of Chemistry and Molecular Engineering, Peking University, Beijing, China; 2 Department of Biomedical Engineering, Stony Brook University, Stony Brook, New York, United States of America; University of Houston, United States of America

## Abstract

Organic solvents offer a new approach to formulate DNA into novel structures suitable for gene delivery. In this study, we examined the *in situ* behavior of DNA in *N*, *N*-dimethylformamide (DMF) at low concentration via laser light scattering (LLS), TEM, UV absorbance and Zeta potential analysis. Results revealed that, in DMF, a 21bp oligonucleotide remained intact, while calf thymus DNA and supercoiled plasmid DNA were condensed and denatured. During condensation and denaturation, the size was decreased by a factor of 8–10, with calf thymus DNA forming spherical globules while plasmid DNA exhibited a toroid-like conformation. In the condensed state, DNA molecules were still able to release the counterions to be negatively charged, indicating that the condensation was mainly driven by the excluded volume interactions. The condensation induced by DMF was reversible for plasmid DNA but not for calf thymus DNA. When plasmid DNA was removed from DMF and resuspended in an aqueous solution, the DNA was quickly regained a double stranded configuration. These findings provide further insight into the behavior and condensation mechanism of DNA in an organic solvent and may aid in developing more efficient non-viral gene delivery systems.

## Introduction

The main hurdle for gene therapy is the delivery of the DNA into target cells in an efficient, safe and bioactive manner. Non-viral vectors such as functionalized cationic lipids or polymers that can condense DNA show great advantages over viruses in terms of safety and low immunogenicity [1]. However, the transfection efficiency of non-viral vectors is lower than achievable with viruses, and is closely related to the specific structure formed by the DNA interaction with cationic lipids or polymers [2], [3], [4]. Non-aqueous solutions offer a practical approach to create DNA complexes with novel structure and properties. For example, plasmid DNA or oligonucleotides were successfully encapsulated in cationic liposomes with diameter about 100 nm in the presence of 25∼100% ethanol [5], [6], [7], [8], [9]. Other organic solvents have also been used for a similar purpose [10], [11], [12], [13]. Moreover, we have previously demonstrated the encapsulation of plasmid DNA in 94% dimethylformamide (DMF) and its processing into biodegradable poly(lactide acid)/poly(glycolide acid) (PLGA) nonofibers via electrospinning [14], [15]. The condensed conformation of the DNA chain in DMF was demonstrated by LLS and visualized by TEM [15].

Even though organic solvents have been used to manipulate non-viral gene vectors into novel structures, the integrity and dynamics of DNA molecules in organic solvents has not been truly elucidated. It is generally believed that the organic solvent screens the electrostatic repulsion between DNA chains and condenses DNA mainly by excluded volume interactions [16], [17], [18]. Further, precipitation of DNA occurs in most of the organic solvents. For example, Mel'nikov et al. [19] studied the discrete phase transition of single DNA chains from an elongated coil to a compacted globule by fluorescence microscopy in aqueous mixtures with a variety of organic solvents, including primary alcohols, acetone and ethylene glycol. The study concluded that the dielectric constant of the solvent was a key factor in determining the conformational behavior of an isolated DNA chain in solution. In solvents with a low dielectric constant, the Coulombic interaction was increased, and the disassociation of counterions in DNA was limited. The authors attributed the attraction to the increased ion-ion correlation interactions [19].

Besides conformation, the integrity and bioactivity of the DNA is also a key issue upon condensation. It is usually considered that DNA maintains its helical structure in the condensed state [16], [20], [21], [22], [23]. However, this is not always the case. Bonner and Klibanov investigated the structural stability of DNA molecules in mixed solvents of 1% water with 99% glycerol, formamide, methanol and DMSO, separately [24]. The data derived from UV absorbance indicated that the double-helix structure was only maintained in 99% glycerol, suggesting that the hydrophobic interactions destabilized the double-helical structure of DNA. More recently, using single molecule atomic force microscopy and molecular dynamic simulations, Cui et al. confirmed that the secondary structure was destroyed when double stranded DNA was placed into a poor solvent such as 1-propanol [25]. Further, Mikhailenko et al. investigated the condensation of individual large DNA molecules in tert-butanol at varying temperatures and found that the denaturation of DNA was highly linked with the unfolding transition [26].

Thus, further elucidation of the structure and integrity of DNA in organic solvents and its effect on transfection efficiency will pave the road to the preparation of novel non-viral vectors for gene delivery. In this work we investigated the integrity of plasmid DNA, calf thymus DNA and an oligonucleotide by a combination of UV absorption, zeta potential analysis, LLS and TEM using DMF as a model solvent as it has been previously used in the preparation of biodegradable scaffolds intended for biomedical applications [14]–[15]. In addition, the structure and bioactivity of DNA was determined after recovery from the solvent. The fact that DNA does not precipitate in DMF at concentrations below 0.1 mg/mL [15], allowed us to study DNA in real time and in a non-disturbing state.

## Results and Discussion

### Loss of Double Stranded DNA Structure as Measured by UV absorption

**Figure 1:**
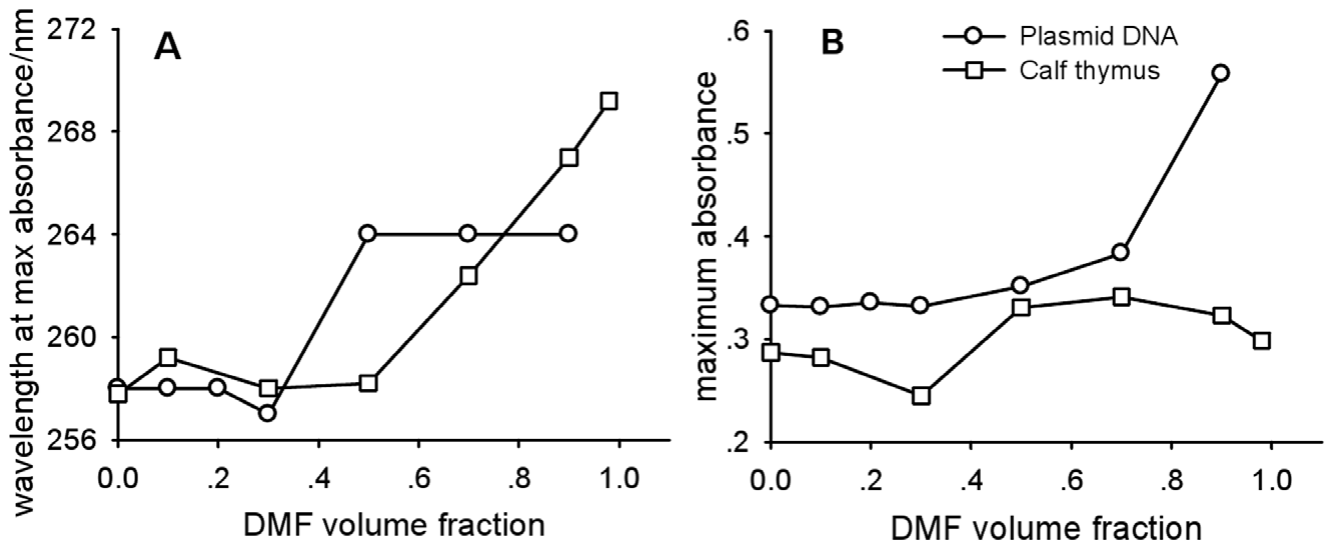
UV absorbance measurements of calf thymus DNA and plasmid DNA (c = 16 µg/mL) in the mixture of TE/DMF at 25°C. A. Wavelength at maximum absorbance/nm. B. Maximum absorbance.

Circular dichroism (CD) conducted at 200–320 nm range was used to determine the helical structure of DNA in an aqueous solution. However, DMF itself shows a strong UV absorbance below 270 nm that renders the CD spectrum of DNA in DMF of no value as a result of this interference. To confirm changes in the double-stranded structure of DNA, we measured the UV absorption of plasmid DNA and calf thymus DNA in a mixed solvent of TE and DMF. It is well established that, upon denaturation, the UV absorbance of DNA is increased due to the hyperchromicity effect, as is the wavelength of maximum absorption. As shown in Fig. 1A, both DNA samples had the same UV wavelength of maximum absorption at 258 nm in an aqueous solution at 25°C. With increasing DMF content to 50 (v/v) %, the UV wavelength of maximum absorption for calf thymus DNA started to increase, and it reached 269 nm at 98% DMF content (Fig. 1A). For plasmid DNA, the wavelength of maximum absorption reached a peak value at 50% DMF and remained constant thereafter (Fig. 1A). As expected, the maximum UV absorbance of calf thymus DNA increased, however, it started to decline when the DMF content reached 70% (Fig. 1B). In contrast, the maximum UV absorbance of plasmid DNA showed a different profile; the absorbance increased steadily with DMF content (Fig. 1B), with a sharp rise at 70% DMF.

**Figure 2:**
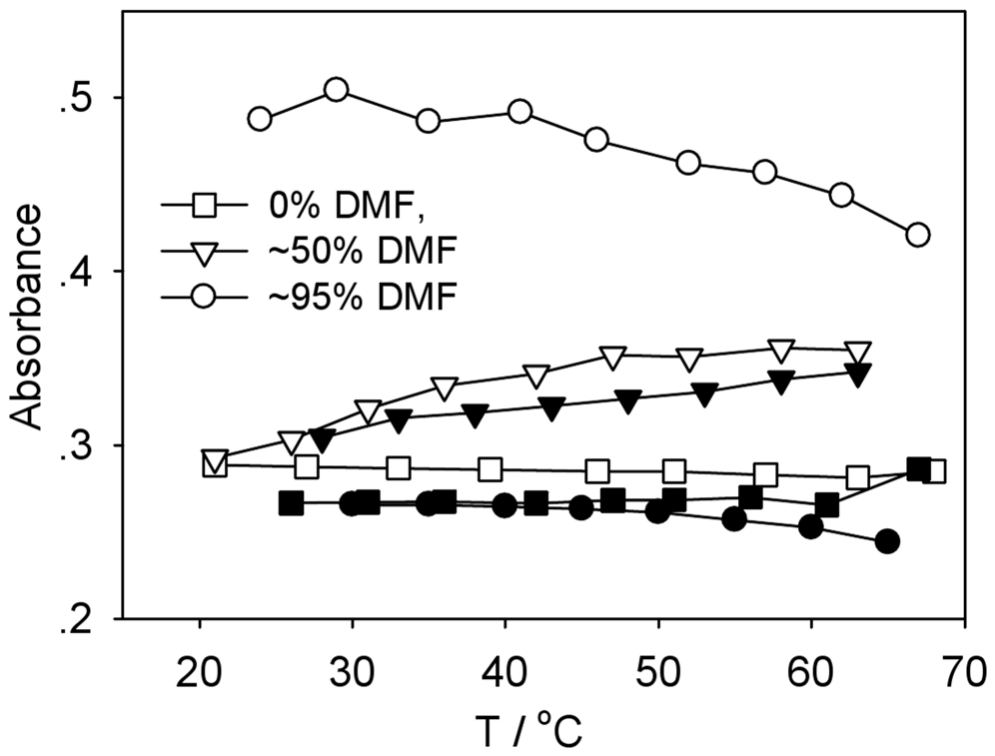
Thermal denature curves of calf thymus and plasmid DNA. Plasmid DNA: hollow symbols; calf thymus DNA: solid symbols. C = 16 µg/mL.

We utilized melting temperature as another test for characterizing DNA denaturation. For dsDNA in aqueous solution, denaturation (the exact melting point is related to the GC content) occurs over a narrow temperature range at above 70°C. Thus, we directly compared the thermal denaturing curves of both plasmid and calf thymus DNA in TE buffer with 0%, 50% and 95% DMF in the temperature range of 20°C–68°C. Without DMF, the melting profiles of both calf thymus and plasmid DNA showed a horizontal line indicating no increase in absorbance since both DNA samples are in a double helical structure at temperatures below 68°C (Fig. 2, square lines). In the mixed solvent with 50% DMF, the absorbance of both DNA samples continuously increased with a rise in temperature (Fig. 2, triangle lines). However, with 95% DMF, a gradual decline in absorbance with increasing temperature was observed (Fig. 2, circle lines), suggesting that the double helix structure was already denatured into single strands before heating, consistent with previous findings [24], [25], [27], [28]. The data also indicated that the initial absorbance of plasmid at 95% DMF was much higher than that of calf thymus DNA at the same concentration (Fig. 2, circle lines). Lastly, another major difference between plasmid and calf thymus DNA at 95% DMF was the drop in absorbance with increasing temperature, where the former lost ∼0.08 in absorbance and the latter ∼0.02 (Fig. 2, circle lines).

**Figure 3:**
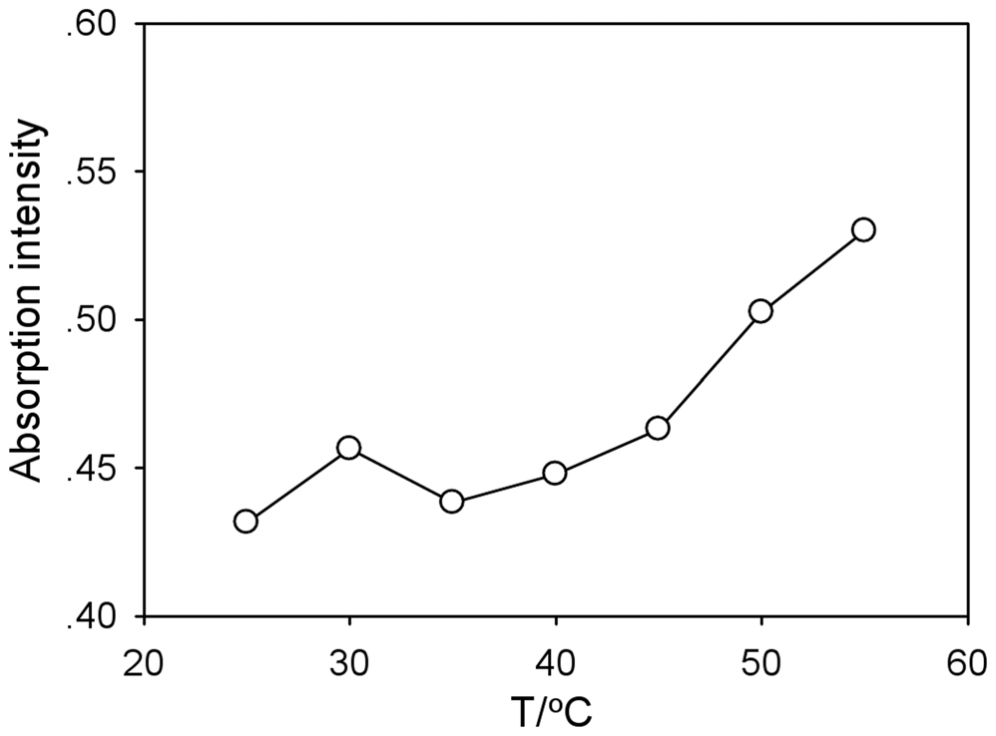
Thermal denature curve of 21bp DNA fragment in 95% DMF. C = 20 µg/mL.

In contrast to the observed effects on both plasmid and calf thymus DNA, the UV absorbance of the 21 bp DNA oligonucleotide in 95% DMF increased with temperature (Fig. 3), but was similar to that in aqueous solution or in 99% glycerol [24], indicating that the 21 bp oligonucleotide was not denatured in the presence of 95% DMF prior to heating.

### Determination of Conformational Changes by Laser Light Scattering

**Figure 4:**
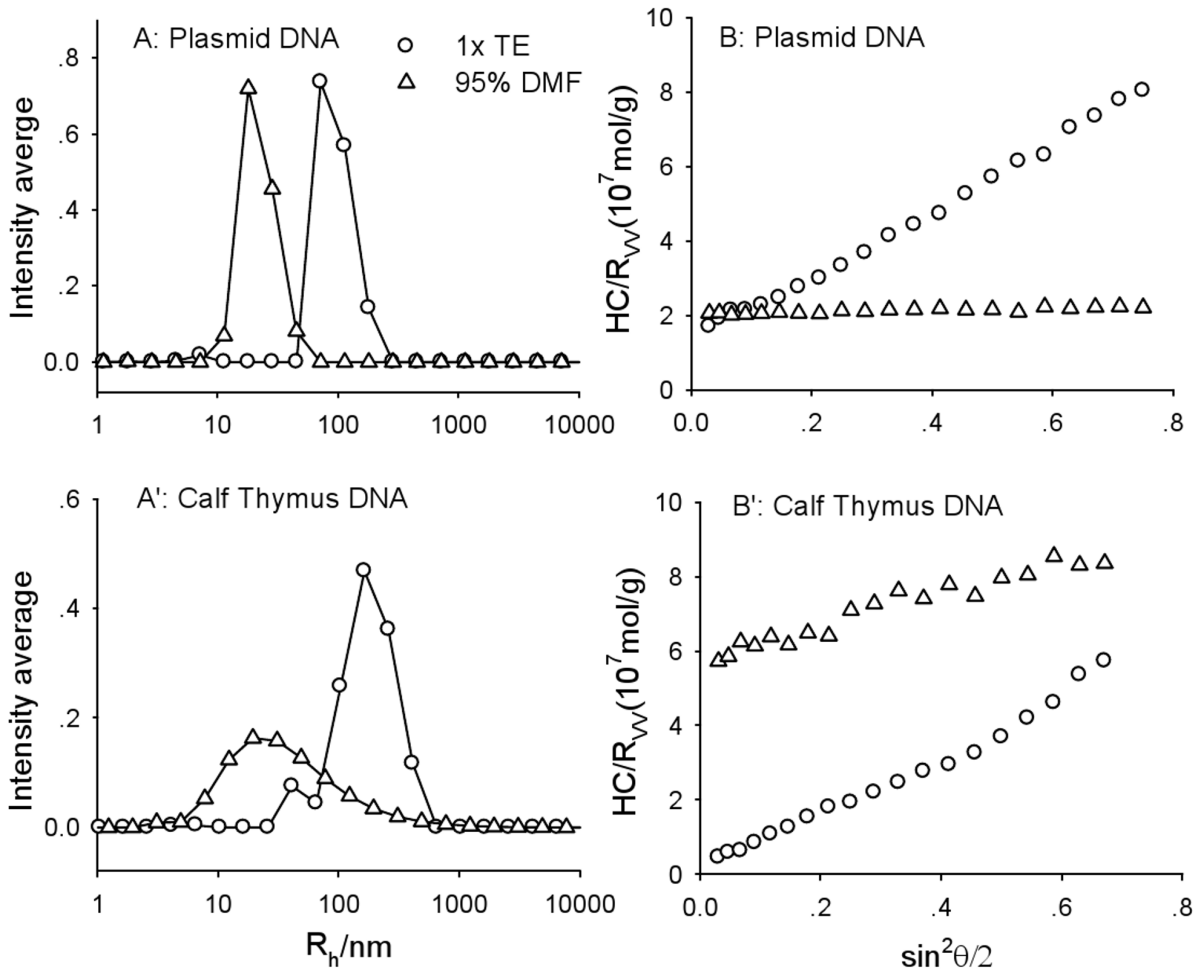
LLS studies on plasmid DNA (A: size distribution at 30°, B: angular dependence of excess scattered intensity) and calf thymus DNA (A': size distribution at 30°, B': angular dependence of excess scattered intensity). C = 1.6×10^−5^ g/ml.

Another possible explanation for the decrease in absorbance with increasing temperature (with 95% DMF, Fig. 2) may be thermo-induced aggregation of DNA in DMF. Thus, we further studied the conformation of plasmid and calf thymus DNA at varying conditions by LLS. Figure 4 shows both the angular dependence of the excess scattered intensity from static light scattering (SLS) and the CONTIN analysis of the correlation curve from the dynamic light scattering (30°) using the DNA samples at 1.6×10^−5^ g/ml at 25°C. The physicochemical parameters of both DNA samples obtained by LLS are summarized in Table 1. Plasmid DNA was different from calf thymus DNA in terms of topology, molecular weight and size distribution. For example, in TE buffer, the M_w,app_ of plasmid DNA as determined by SLS was 7.9 MDa, larger than the actual molecular weight of a DNA sample with 7164 bp, even when considering the effect of concentration (A_2_: ∼8.0×10^−4^mol cm^3^ g^−2^). This difference was probably caused by the insufficient screening of the electrostatic interactions in TE buffer whose ionic strength was not high enough [29]. The determined M_w,app_ of calf thymus DNA was 38 MDa, significantly larger than that of plasmid DNA, as expected. Since its size distribution was quite broad (Fig. 4A), it was difficult to estimate the length of the DNA in base pairs.

**Table 1:** LLS results of plasmid DNA and calf thymus DNA in TE buffer and in 95%DMF.

		R_h,app_/nm	R_g,app_/nm	R_g,app_/R_h,app_	M_w,app_ (10^6^g/mol)
Plasmid DNA	TE buffer	91	161	1.8	7.2
	95% DMF	22	22	1.0	4.9
Calf thymus DNA	TE buffer	154	274	1.8	37
	95% DMF	22	27	1.2	1.6

The conformation of the DNA molecules could be further inferred from determining the R_g_/R_h_ ratio (with R_g_ and R_h_ being the square root of gyration radius and the hydrodynamic radius, respectively). It is well established that the R_g_/R_h_ ratios are 0.775 for a solid sphere and greater than 1.5 for a random coil [30]. As indicated by the R_g,app_/R_h,app_ ratio in Table 1, both, the plasmid and calf thymus DNA samples were in random coil conformation in 1× TE buffer. Further, in 95% DMF, the measured M_w,app_ of plasmid DNA was 4.9 MDa, close to the actual molecular weight of DNA in a double helical state. However, its size was sharply reduced, as shown in Table 1, where the R_g,app_ of the plasmid DNA decreased from 161 nm in TE buffer to 22 nm, a factor of ∼7, while no significant change in the size distribution was observed (Fig. 4A). The R_g,app_/R_h,app_ ratio (∼1.0) also indicated that the plasmid DNA was not in random coil conformation, but in a more condensed state.

The size of calf thymus DNA in 95% DMF was even more condensed, with a decrease of R_g,app_ by a factor of ∼10, from 274 nm to 27 nm. The conformation was also condensed (R_g,app_/R_h,app_ ratio, 1.2),as evident by the broad distribution. As expected, the M_w,app_ of calf thymus DNA in 95% DMF also sharply decreased to 1.6 MDa, an order of magnitude smaller than that in aqueous solution. This is most likely caused by denaturation in DMF, whereby each of the DNA molecules was split into two separated chains, resulting in a decrease in molecular weight. Since the molecular weight determined by LLS was weight-averaged, the broad size distribution (Fig. 4A) dramatically decreased the M_w,app_ of the denatured DNA chains. Another possible reason for the sharp drop in molecular weight may originate from filtration. The DNA chains with extremely large molecular weight were insoluble in DMF, even at low concentrations, and as such they did may not have passed through the filter during sample preparation. As for the plasmid DNA, all molecules possessed the same molecular weight, and supercoiling prevented the chains from being completely separated during denaturation and thus, no sharp decrease in M_w,app_ was observed.

**Figure 5:**
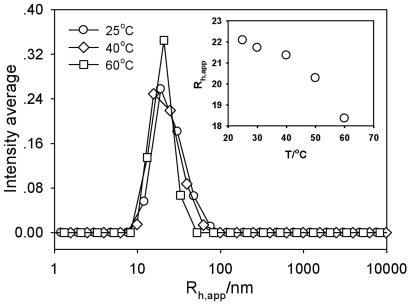
Temperature effect on hydrodynamic radius of plasmid DNA in 95% DMF at 30°. The inset shows the temperature dependence of R_h,app_ at zero angle.

Since we observed a decline in UV absorbance of DNA solutions with increasing temperature, we used plasmid DNA as an example and investigated the effect of temperature on the R_h,app_ in 95% DMF. Results show a decrease from about 22 nm to about 18 nm with increasing temperature from 25°C to 60°C (Fig. 5). SLS showed similar results (data not shown). In addition, the size distribution was also slightly narrowed as a result of varying the temperature. These LLS results support the findings from the UV absorption measurements, where the change in UV absorbance with temperature (Fig. 2) was due to the hypochromicity, not interchain aggregation.

### Verifying DNA Complex Morphology by Transmission Electron Microscopy

**Figure 6:**
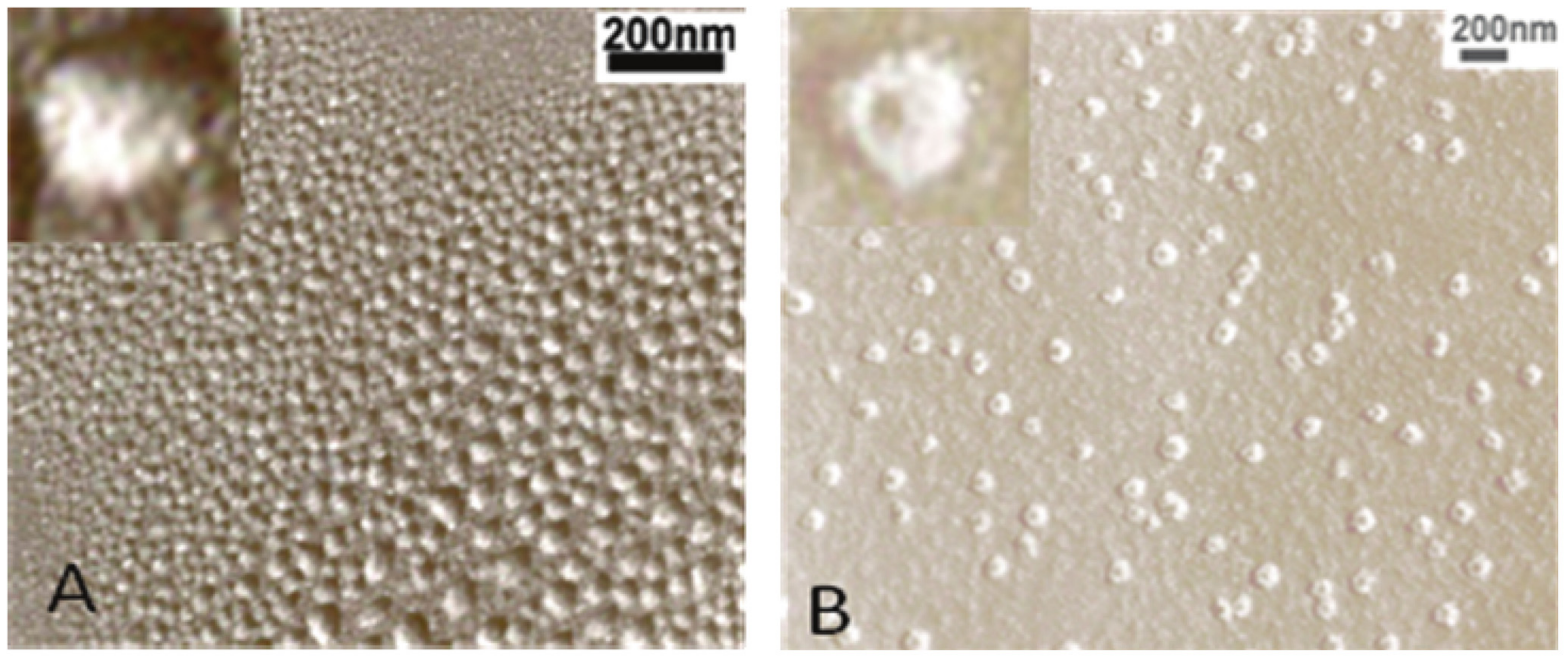
TEM measurements of (A) calf thymus and (B) plasmid DNA air dried from 95% DMF.

In order to verify the morphology of the condensed DNA in 95% DMF, we utilized TEM and imaged plasmid and calf thymus DNA after air drying on a carbon membrane. As shown in Fig. 6A, spherical structures with diameters ranging from 20nm to 60nm were observed for calf thymus DNA, in agreement with the LLS results. For plasmid DNA, toroid-like structures with inner- and outer diameters of ∼30nm and ∼100nm, respectively, were observed (Fig. 6B). The size distribution of these toroid-like structures was quite uniform, suggesting that they were formed by single DNA chains. The growth of such toroid-like structures from semiflexible chains, especially dsDNA in the presence of multivalent cations, has been reported in the literature [31], [32], [33], [34]. For example, it was previously proposed that toroid conformation was formed through a process of nucleation and growth [31], [33]. We speculate that with increasing DMF content, DNA chains underwent both denaturation and condensation. The condensation resulted mainly from the limited disassociation of the counterions and the enhanced excluded volume interactions. Upon denaturation and condensation, some of the intertwined points in the plasmid DNA could act as nucleation sites and initiate the growth of a toroid. Since each plasmid DNA has more than one intertwined point and these points are not uniformly distributed, the observed toroid-like structures were not necessarily symmetrical, where the inner hole was not centered and the contour was not smooth, as observed (Fig. 6B, inset). As for the calf thymus DNA, the denatured DNA strands were quite flexible and followed a different pathway to form globules [35], [36], [37]. The size of the plasmid DNA in 95% DMF as determined by TEM was twice larger than that determined by LLS, which was probably due to the collapse of the toroid structure on the surface. Recently, Chang et al. reported the R_g_/R_h_ value of toroid micelles was about 1.0 [38], indicating that toroid structures indeed exist in solution, not only as surface-directed [39], [40].

### DNA Condensation and Charge Density as Determined by Zeta Potential

**Figure 7:**
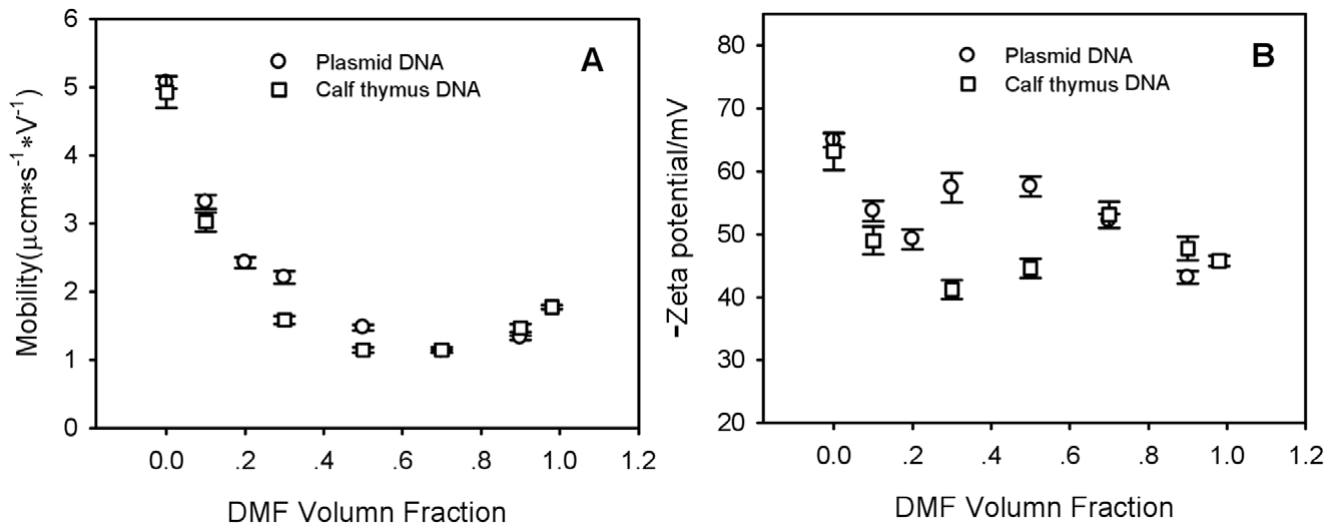
DMF-content dependence of zeta potential measurements of calf thymus DNA and plasmid DNA.

Effective charge density is a crucial parameter in determining the structure and morphology of DNA in a condensed state. It is generally considered that DNA condensation occurs when ∼90% of charge is neutralized in an aqueous solution [41]. This may not be the case in organic solvents. Our results show the mobility and the corresponding zeta potential of both plasmid and thymus calf DNA samples in the mixed solvent with varying DMF content. For example, at 50% DMF, the mobility of both DNA samples decreased sharply by a factor of ∼5 (Fig. 7A). Further addition of DMF resulted in slightly increased mobility (Fig. 7A). The zeta potential was calculated according to Equation 2 (Materials and Methods) and as shown in Fig. 7B, it was decreased from ∼64 mV in 1xTE buffer to ∼45 mV in 95% DMF for both DNA samples. Even though it was difficult to obtain the exact value of surface charge via zeta potential, especially in a mixed solvent, we reasonably conclude that the condensed DNA in 95% DMF was partially disassociated into polyions. The dielectric constant of DMF is 36.7 at 25°C, between those of water (78.5) and most other organic solvents. The dipole moment of DMF is 3.82 D, much larger than that of water (1.85D). Therefore, the dissociated counterions are energetically stable in DMF, leaving the condensed DNA negatively charged. In 95% DMF+5% TE buffer, the Debye screen length 1/κ was estimated to be ∼20 nm according to the equation [42]

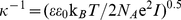
(1)with ε, ε_0_, e, *I* being the solvent dielectric constant, the dielectric constant in vacuum, the elementary charge, the ionic strength of the solution, respectively. This indicated that the intra or inter chain electrostatic repulsion was quite strong, and prevented the formation of strong aggregates. As a result, condensation of single chains occurred.

### Denaturation of DNA in Organic Solvent

The aforementioned measurements offered a clear picture on the behavior of DNA in an organic solvent. Larger size DNA was condensed and denatured, while smaller size DNA was basically intact. The critical size was probably related with the persistence length of DNA and the nature of solvent. In DMF, the condensed and denatured DNA has the R_g_ about 20 nm (Table 1), therefore, the smaller size DNA should remain structurally intact. Besides DNA size, the content of organic solvent is also a key parameter in determining the degree of denaturation. The UV absorbance data shown in Fig. 1 indicated that DNA was denatured when the DMF content reached a certain value; at 50% DMF, the double strand structure was preserved but it was not stable, and it was subject to temperature change (Fig. 2). For calf thymus DNA, denaturation was completed at ∼70% DMF (Fig. 1B) and since DMF is a poor solvent, additional DMF resulted in a more condensed DNA conformation (Fig. 1), thus, leading to a decrease in absorbance (Fig. 1). For plasmid DNA, denaturation was complete at DMF content below 95%, however, the two single strand chains intertwined together, and the intrinsic torsional tension related with the supercoiled topology rendered the denatured plasmid DNA in a more condensed conformation different from that of calf thymus DNA (Table 1 and Fig. 4). Therefore, its absorbance was higher than that of calf thymus DNA at greater DMF content, and it did not reach a maximum value. The condensed DNA was also further reduced in size with increasing temperature, leading to a decrease in absorbance due to a hypochromic effect.

The condensation of DNA observed in organic solvents was different from that induced by multivalent counterions or polyions in aqueous solutions, mainly as driving forces. In aqueous solution, DNA was condensed when the charge was neutralized by more than 90%, and the attractive force was attributed to the ion-ion correlation interactions [43]. While in organic solvent, Monte Carlo simulation conducted by Mel'nikov' et al showed that the polymer chains shrank with decreasing dielectric constant and indicating that ion-ion correlation also played a role in DNA condensation [19]. However, our measurements (Fig. 7) indicated that the negative charges of DNA in DMF were strong enough to prevent the chains from being condensed. From the viewpoint of polymer physics, we attributed the extra attractive force to the short-ranged excluded volume interactions. Since DMF was a poor solvent for DNA, the excluded volume was decreased with increasing DMF content, resulting in the collapse of the DNA chains. Since the intrinsic persistence length of DNA was ∼50 nm [44], which was even larger considering the contribution arising from electrostatic repulsions, it prevented the DNA from condensing into particles with smaller diameters. One possibility for achieving this was to denature the DNA into single strands whose persistence length was shorter than 2nm. Besides this shorter length, the hydrophobicity of ssDNA was also stronger, further reducing the free energy by remaining as such in the organic solvent. The ssDNA conformation in a condensed state has also been experimentally shown by Osada et al. [45]. Since the solubility in DMF was improved with decreasing DNA size, no denaturation could occur when the DNA size was smaller than a certain value.

### Renaturation of Plasmid DNA Upon Recovery to Aqueous Solution

**Figure 8:**
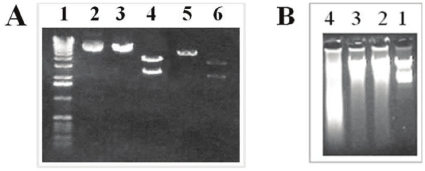
Agarose gel electrophoresis of DNA samples. (A) Plasmid DNA: M_W_ marker (lane 1), control DNA (lane 2), control DNA cut with *Eco*RI, which linearized the plasmid but would not alter the size (lane 3), control DNA cut with *Pst*I showing 2 fragments with different lengths (lane 4), DNA treated with DMF and cut with *Eco*RI (lane 5) or *Pst*I (lane 6); (B) Calf thymus DNA: M_W_ marker (lane 1), control DNA (lane 2), DNA treated with water (lane 3), DNA treated with DMF (lane 4).

To investigate the recovery of the denatured structure, we exposed calf thymus and plasmid DNA to 95% DMF overnight at room temperature. The solvent was removed by evaporation, and the DNA was reconstituted in TE buffer. As shown in Figure 8A, plasmid DNA treated with DMF exhibited the same mobility as the control, following restriction enzyme digestion, indicating that the double-stranded structure of plasmid DNA was intact. Moreover, the restriction enzymes used, *Eco*R1 and *Pst*1, were able to cut the DMF-treated plasmid DNA into the exact same fragments as that of the control, suggesting that the nucleotide sequence of the plasmid DNA was not altered after exposure to DMF. This experiment suggested that the double-stranded structure of plasmid DNA rapidly recovered in TE buffer. In contrast, calf thymus DNA did not rapidly recover after being resuspended in an aqueous solution for one day, and as shown in Figure 8B, the DNA treated with DMF appeared as both a band and a smear (lane 4), indicating that the denatured single stranded calf thymus DNA did not renature completely.

## Materials and Methods

### Reagents

pCMVβ plasmid DNA (7164 bp) encoding β-galactosidase (Clontech, Palo Alto, CA) was purified from cultured *E.coli* utilizing a Qiagen (Valencia, CA) DNA isolation kit. Calf thymus DNA was purchased from Sino-American Biotechnology Company (Beijing, China). Gel electrophoresis verified that the calf thymus DNA contained fragments ranging from several hundred bps to several tens of thousands bps (data not shown). A 21bp oligonucleotide with random sequence and purity >90% was purchased from SBS Genetech Co. LTD (Beijing China). All DNA samples were prepared in 1×TE buffer (10mM Triz base, 1mM EDTA). The concentration of the stock solution of all samples was higher than 1.5 mg/mL. The A_260_/A_280_ ratios of calf thymus DNA and plasmid DNA solutions were ∼1.8, indicating no protein or RNA contamination. During the experiments, stock solutions of each DNA sample were diluted with either TE buffer or DMF to known concentrations. HPLC grade DMF was purchased from Beijing chemical company (Beijing, China) and used as received. Milli-Q water (18.2 MΩ·cm) was used in all experiments.

### UV absorbance

The UV absorbance of the DNA samples at 16.0 µg/mL was scanned from 200 nm to 600 nm on a Shimadzu 2101 UV/Vis spectrometer. The temperature was controlled by a water bath (Polyscience, USA) with the precision at 0.1°C. The solvent was used as the control.

### Laser Light Scattering (LLS)

A commercial laser light scattering spectrometer (Brookhaven Inc, Holtsville, NY) equipped with a BI-200SM goniometer and a BI-TurboCorr digital correlator was used to perform both static light scattering (SLS) and dynamic light scattering (DLS) over scattering angles ranging from 20° to 120°. A 100 mW, vertically polarized solid state laser (GNI, Changchun GXC-III, 532 nm) was used as the light source. In static light scattering, the angular dependence of the excess absolute time-averaged scattered intensity, known as the Rayleigh ratio *R*_vv_(*θ*), was measured. For a very dilute solution, the weight-averaged molar mass (*M*_w_) and the root mean-square radius of gyration (*R*_g_) can be obtained on the basis of
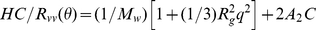
(2)where *H* = 4π^2^ *n* ^2^(d*n*/d*C*)^2^/(*N*_A_ *λ* ^4^) and *q* = 4π*n*/*λ* sin(*θ*/2) with *N*_A_, *n*, d*n*/d*C*, and *λ* being the Avogadro's number, the solvent refractive index, the specific refractive index increment, and the wavelength of light in a vacuum, respectively. The dn/dc values of DNA in aqueous solution and in DMF were obtained from the literature [15]. In dynamic light scattering, the intensity–intensity time correlation function G^(2)^(t) in the self-beating mode was measured. It is related to the normalized first-order electric field time correlation function g^(1)^(t). A Laplace inversion program, CONTIN, was applied to analyze g^(1)^(t) to obtain the hydrodynamic radius, R_h,app_, and its distribution.

### TEM

5 µL of the DNA solution (10 µg/mL) was placed on a pure-carbon membrane and left at room temperature for ∼12 h. The samples were then coated with a film of Pt by electrospray at an angle of 45°. TEM images were taken by using JEM-200CX transmitting electron microscopy (JEOL, Japan) at an accelerated voltage of 120 kV.

### DNA digestion and gel electrophoresis

10 µg of plasmid DNA in TE was lyophilized in a speed vacuum and then resuspended in 95 µL DMF and 5 µL TE buffer (5% aqueous solution) and kept overnight at 4°C. The following day 10 µL of the dissolved plasmid (1µg) was again lyophilized to remove the solvent, and then resuspended in 4 µL TE buffer for plasmid digestion with either *Eco*RI (2 µL) or *Pst*I (2 µL) for 4 h at 37°C followed by gel electrophoresis. The resulting DNA fragments were visualized by ethidium bromide staining. For the plasmid utilized in the study, there is a single *Eco*RI restriction site (simply linearizes the plasmid) while two restriction sites are present for *Pst*I (generating two fragments). Similarly, 1 µL calf thymus DNA stock solution (c = 1.67 mg/mL ) was added to 9 µL water or DMF and kept overnight. Then the solutions were lyophilized in vacuum and dissolved in 1 µL water+4 µL TE for gel electrophoresis. The control sample was diluting the stock solution to 4 µL TE directly. The integrity of calf thymus DNA was also analyzed by gel electrophoresis.

### Zeta potential measurements

A Zeta potential analyzer from Brookhaven Instruments Corporation (ZetaPALS, Holtsville, NY) was used to measure the Zeta potential of the DNA samples in different solvents at 25°C. The Palladium electrode ensured the validation of the measurement in DMF. The mobility (μ_e_) of DNA molecules was determined by the analyzer via Phase Analysis Light scattering (PALS). The zeta potential ζ was calculated according to:
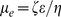
(3)with ε and η being the dielectric constant and the viscosity of the solvent, respectively.
